# Valley-engineered ultra-thin silicon for high-performance junctionless transistors

**DOI:** 10.1038/srep29354

**Published:** 2016-07-08

**Authors:** Seung-Yoon Kim, Sung-Yool Choi, Wan Sik Hwang, Byung Jin Cho

**Affiliations:** 1 School of Electrical Engineering, KAIST, Daejeon, 305-701 Korea; 2 Department of Materials Engineering, Korea Aerospace University, Gyeonggi-do, 412-791, Korea

## Abstract

Extremely thin silicon show good mechanical flexibility because of their 2-D like structure and enhanced performance by the quantum confinement effect. In this paper, we demonstrate a junctionless FET which reveals a room temperature quantum confinement effect (RTQCE) achieved by a valley-engineering of the silicon. The strain-induced band splitting and a quantum confinement effect induced from ultra-thin-body silicon are the two main mechanisms for valley engineering. These were obtained from the extremely well-controlled silicon surface roughness and high tensile strain in silicon, thereupon demonstrating a device mobility increase of ~500% in a 2.5 nm thick silicon channel device.

Wearable electronics have received huge attention as the next wave of electronic products. The demand for wearable or flexible electronic devices has encouraged researchers to look into two-dimensional (2D) materials such as graphene and transition metal dichalcogenide (TMD) materials such as molybdenum disulfide^1^,^2^,^3^,^4^. These materials can be used as channel materials for field effect transistors (FET) or sensors by exploiting their thin nature. Graphene, the representative 2D material, shows high mobility however it could be used in limited application due to its zero bandgap property^1^,^2^. TMD materials such as MoS_2_ could be a good candidate to demonstrate flexible electronics and it shows reasonable device performance^3^. However, TMD channel devices have shown inferior performance than silicon channel devices in terms of sub-threshold swing, channel mobility and interface state density. Black phosphorus has a great potential for the future electronics which shows acceptable on/off ratio and high mobility^5^. However, material synthesis in a large scale with good uniformity as well as the low defect density required for electronic device applications is still a challenge. Silicon can provide a practical solution to circumvent such problems, as an industrially mature technology is in place for silicon based electronics^6^,^7^,^8^,^9^. Extremely thin silicon show good mechanical flexibility because of their 2-D like structure and enhanced performance by the quantum confinement effect^10^,^11^,^12^,^13^,^14^,^15^. Here, we experimentally demonstrate a junctionless FET revealing a room temperature quantum confinement effect (RTQCE) by using a valley-engineered 2D-like silicon. Strain-induced band splitting^16^,^17^,^18^ and a quantum confinement effect induced from ultra-thin-body silicon^19^,^20^,^21^ are the two main mechanisms for valley engineering. The term “valley engineering” has been used to express the control of sub-band separation energy to achieve higher performance of silicon device^22^,^23^. Low effective mass of the carrier in the transport direction is achieved, together with suppression of scattering-inducing components, resulting in enhanced device performance. These advantages were obtained by careful control of the silicon surface roughness and complexity, and high tensile strain in silicon, thereupon demonstrating a device mobility increase of ~500% in a 2.5 nm thick silicon channel device.

## Results

In order to make ultra-thin-body silicon, a thinning process was conducted by repetitive low temperature oxidation of silicon at 750 °C, followed by wet chemical etching of the oxide. Silicon was thinned to 2.5, 5, and 7 nm. The surface of the silicon was carefully controlled so that root mean square (RMS) surface roughness of around ~0.13 nm could be achieved. Tight control of the surface roughness is important to maintain the 2D density of state (DOS) of silicon since a RTQCE can only be observed in ultra-thin-body silicon with a sharp DOS when the RMS surface roughness is smaller than 0.4 nm^10^.

**Figure 1:**
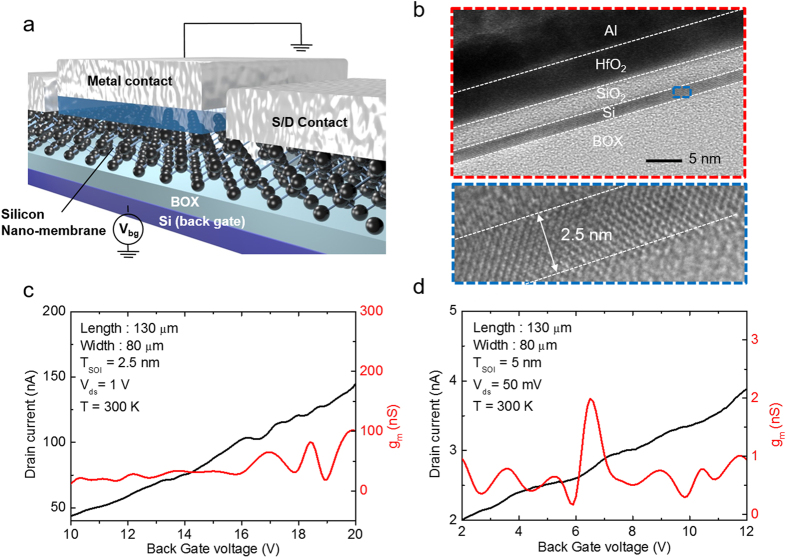
(**a**) Schematic drawing of the device structure. (**b**) TEM image of the fabricated device. It shows an ultrathin silicon layer with excellent thickness uniformity. The magnified image shows a 2.5 nm thick single crystal silicon. (**c**) Transfer characteristics of a silicon transistor when the silicon thickness is 2.5 nm. The drain current shows step-like increments and the trans-conductance is oscillated, implying RTQCE. (**d**) Transfer characteristics of a silicon transistor when the silicon thickness is 5 nm. The device also reveals RTQCE.

Figure 1a,b respectively present a schematic drawing and a high-resolution TEM image of the fabricated silicon junctionless FET. The TEM image shows that a 2.5 nm silicon is uniformly formed in the channel region. The device is designed to be operated using a back gate electrode as the main controlling gate, while the front gate stack (metal electrode and high-K gate dielectric) is used only for passivation, and for this the front gate is grounded during device operation. Such operation is meant to minimize the effect of the access resistance that exists due to the physical gap between the front gate and the source/drain region where the silicon resistance is not modulated by the front gate. The accumulation mode conduction mechanism by adopting a junctionless FET structure was used. The silicon was moderately n-type doped, for both the channel and source/drain region. Figure 1c,d show the transfer characteristics of the fabricated device under a bias of V_D_ = 1 V at room temperature when the body thicknesses is 2.5 nm and 5 nm, respectively. Step-like increments of the drain current are observed throughout the whole sweep range of the gate voltage. Oscillation of the trans-conductance is clearly observed, indicating that RTQCE occurs. Since the body thicknesses of the devices are thinner than the thermal de Broglie wavelength of silicon at room temperature, that is, 12 nm, RTQCE is clearly observable^10^.

**Figure 2:**
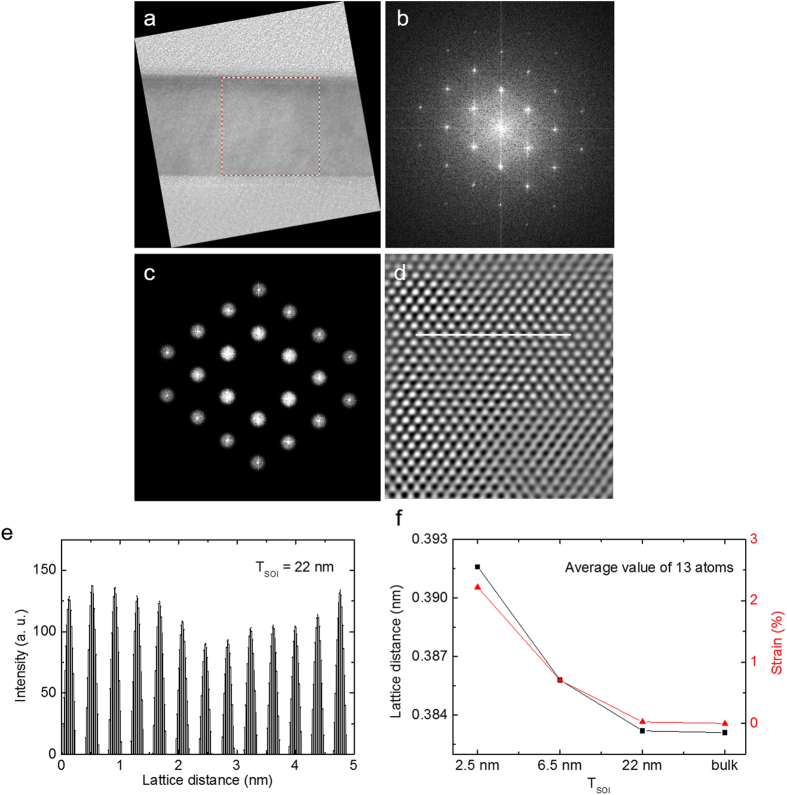
(**a**) High-resolution TEM image of a 22 nm thick top silicon. (**b**) FFT image (**c**) A mask was applied to the FFT image to utilize the information. (**d**) A reconstructed inverse FFT image was used to analyze the lattice distance in the silicon. (**e**) Intensity of lattice distance from the inverse FFT image. (**f**) Extracted lattice distance versus silicon thickness. The calculated tensile strain along the transport direction is plotted together against the silicon thickness.

To investigate the governing mechanism of RTQCE in the silicon, the lattice structure of the ultra-thin silicon was carefully analyzed using TEM. The ultra-thin silicon shows up to 2.2% tensile strain when the thickness of the silicon is thinned to 2.5 nm. This high tensile stress is attributed to our thinning process, which involves repetitive low-temperature (750 °C) oxidation of silicon and stripping the silicon dioxide layer. Silicon dioxide formed by the low-temperature oxidation process has high viscosity. Stress caused by the oxidation process is proportional to the product of the growth rate and oxide viscosity. Successive oxidation below the glass transition temperature, which is 960 °C, results in high biaxial tensile strain without viscoelastic relaxation^24^,^25^. Tensile strain is also in the transport direction because strain is generated biaxially. Strain in silicon was quantified through the sequence shown in Fig. 2a,d. First, high-resolution TEM images of 2.5 nm, 6.5 nm, and 22 nm thick silicon were obtained and an image of the bulk silicon was captured as a reference. A Fast Fourier Transform (FFT) was conducted, followed by applying a mask to the FFT image. To reconstruct the atomic arrangement for calculating the lattice distance, an inverse FFT was conducted. The lattice distance was acquired via the white line in Fig. 2d and was revealed to be 0.3832 nm for the 22 nm thick silicon. The same process was performed for the 2.5 nm and 6.5 nm thick silicon, as well as the bulk silicon, yielding a lattice distance of 0.3916 nm, 0.3858 nm, and 0.3831 nm, respectively. As a result, 2.2% tensile stress in [110] the transport direction was measured in the 2.5 nm thick silicon. This amount of strain in the silicon is much higher than that of the commercial strained silicon wafer by using a SiGe strained layer (about 1%)^26^. Both the nature of the ultra-thin-body (2.5 nm) and the high tensile strain (2.2%) lead to splitting valleys of the silicon energy band, which suppresses inter-valley scattering. In addition, this strain-induced valley splitting also has advantages in terms of maintaining low transport mass since most electrons are occupied in a two-fold valley of the strained ultra-thin-body silicon at room temperature^8^,^19^.

**Figure 3:**
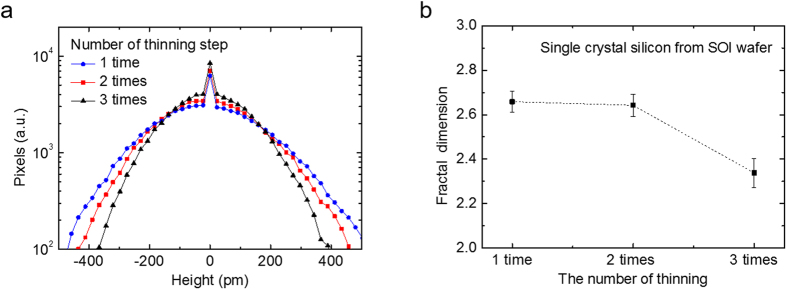
Surface roughness measured by non-contact mode AFM. (**a**) Height histogram graph of the thinned sample with respect to the number of iterations of the silicon thinning process. The distribution of heights tends to narrow as the thinning cycle increases. In order to clearly show the difference in the height histograms, the y-axis uses a log scale. (**b**) The fractal dimension of thinned samples is calculated by XEI, showing the complexity and flatness of the surface. The three-times-thinned sample shows the lowest value, 2.338, which implies that its flatness level is similar to a 2-D material.

Figure 3 shows the surface roughness measured by non-contact mode AFM. AFM was performed on the top surface of thinned silicon on a SOI wafer over an area of 10 μm × 10 μm. The RMS values of the surface roughness after 1, 2, and 3 iterations of the thinning process (low temperature oxidation and stripping the oxide) are 0.164 nm, 0.153 nm, and 0.138 nm, respectively, which are almost identical to the RMS roughness of graphene, a 2D material^27^. The height histogram of the thinned samples is shown in Fig. 3a. As the number of thinning processes increases, the RMS roughness tends to slightly decrease, which is correlated with the strain, as higher strain causes a smoother surface^28^. As the RMS roughness slightly decreases when the number of thinning iterations increases, the fractal dimension, which indicates the flatness and complexity of the surface, decreases to 2.338. This high level of flatness indicates that it could be an excellent choice for a 2D-like substrate for high performance electronic devices.

**Figure 4:**
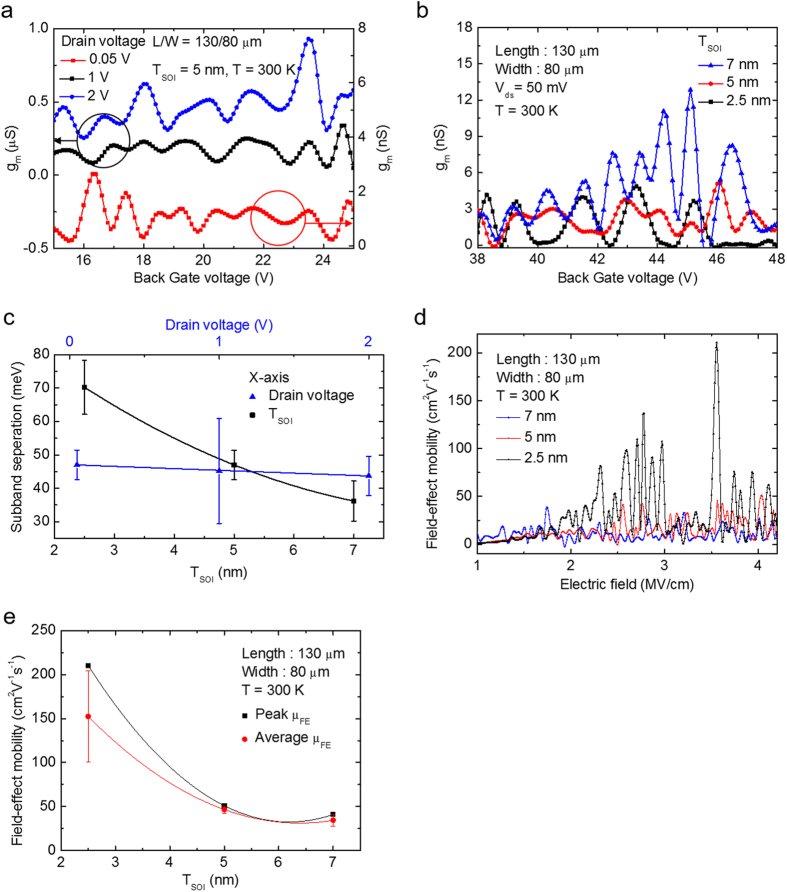
(**a**) Trans-conductance oscillation for different drain voltages. Room temperature quantum confinement (RTQCE) is observed regardless of the drain voltage. The inter-peak voltage indicates the amount of sub-band separation. (**b**) Conductance oscillation was clearly shown when the top silicon thickness is below 7 nm. The voltage interval between peaks clearly increases as the top silicon thickness decreases. (**c**) Calculated sub-band separation. 70.2 meV valley splitting was obtained in the 2.5 nm thick device. Since valley splitting is greater than the Brillouin zone end phonon energy, at about 60 meV, inter-valley phonon scattering is suppressed, which directly affects the mobility. (**d**) Field effect mobility versus electric field. (**e**) Field effect mobility versus top silicon thickness. A fivefold increment in field effect mobility was obtained in the 2.5 nm thick silicon device compared to that of the 7 nm thick device, as the amount of sub-band separation in the 2.5 nm thick silicon channel device is larger the typical Brillouin zone end phonon energy.

Further analyses of the electrical characteristics of valley-engineered silicon transistors are presented in Fig. 4. As mentioned earlier, in this measurement, the top gate electrode on the HfO_2_ passivation layer was grounded for accurate measurement^29^. Buried oxide (BOX) underneath the silicon was used as a gate dielectric, the thickness of which was 140 nm. Figure 4a shows the drain voltage (V_D_) dependence of the conductance oscillation for V_D_ = 0.05 V, 1 V, and 2 V. The average peak-to-peak voltage in conductance oscillation shows no clear correlation to the drain voltage. However, the conductance oscillation has a strong and clear dependence on the silicon thickness, as shown in Fig. 4b. As the silicon thickness decreases, the inter-peak voltage is enlarged. These results are expected and also evidence that the oscillation is from the sub-band splitting, because the drain voltage does not affect sub-band splitting whereas the decrease of the silicon thickness will increase the sub-band separation. Sub-band separations, the amount of valley splitting, were calculated using the measured inter-peak voltage. (See Supplementary Information Part 3) Fig. 4c shows the calculated sub-band separation with respect to the drain voltage and silicon thickness. The amount of sub-band separation in the 2.5 nm thick silicon channel device is 70.2 meV, which is larger than the typical Brillouin zone end phonon energy (~ 60 meV in Si)^10^. This large amount of sub-band separation is critical because the inter-valley scattering in narrow valleys is greatly suppressed when valley splitting is larger than 60 meV. The extracted field effect mobility in Fig. 4d,e also verifies the relation between mobility and sub-band separation. When, calculating mobility, the transconductance was used when V_ds_ = 0.05 V. In mobility versus gate voltage, the mobility curve has several peaks because of the conductance oscillation caused by quantum confinement effect. The peak mobility in Fig. 4e indicates the mobility value of the highest peak, while the average mobility in the same figure means the average value taken from the top 5 highest peaks. The reason to plot the average mobility together with the peak mobility is to ensure the general trend of the mobility behavior against silicon thickness variation. The mobility of the 2.5 nm thick silicon channel device shows a sudden increase, and is almost 5 times higher than that of the 7 nm thick silicon channel device. The mobility of the 5 nm and 7 nm thick silicon channel devices is similar at around 50 cm^2^V^−1^s^−1^. The main reasons of low mobility for these silicon thicknesses are the junctionless mode of device operation mechanism and high resistance because of ultra-thin silicon thickness. High channel doping of junctionless transistor results in lower mobility compared to inversion mode transistor. High series resistance in S/D region also contributed to the low mobility. Although the amount of sub-band separation increases almost linearly as the body thickness decreases, the mobility increases sharply only in the 2.5 nm thick silicon channel device. This result confirms that the sub-band separation in 2.5 nm thick silicon is indeed larger than the Brillouin zone end phonon energy (60 meV in silicon). The combination of these two mechanisms – a quantum confinement effect due to nanoscale thickness and a strain effect – lead to enhanced performance via engineering valleys in the silicon.

In summary, valley engineering of silicon was conducted by a silicon thinning process using repeated low-temperature oxidation and stripping the oxide. By carefully controlling the silicon surface it was possible to achieve an extremely smooth surface, reaching an almost single atomic level roughness of ~0.13 nm. The repeated low temperature oxidation achieved a high tensile strain of 2.2% in silicon channel. These techniques result in larger sub-band separation in the 2.5 nm thick silicon channel device than the Brillouin zone end phonon energy. The results presented in this paper demonstrate that transistor performance can be dramatically enhanced by the valley engineering of silicon together with RTQCE. These findings are expected to contribute to the advancement of low dimensional silicon device technology, which can open a pathway for realizing high performance flexible electronic devices with strong potential for commercialization.

## Methods

### Silicon preparation and device fabrication

The silicon was fabricated by starting with a commercially available silicon-on-insulator (SOI) wafer. The SOI thickness of the starting wafer was 100 nm. First, high-temperature furnace oxidation at 1000 °C and stripping the oxide were repeated until the SOI thickness reached approximately 10 nm. Low-temperature oxidation at 750 °C for 25 min and stripping the oxide were then repeated until the SOI thickness decreased to 7 nm, 5 nm, or 2.5 nm. Each low oxidation cycle can remove silicon thickness of 2.5 nm. To fabricate a junctionless transistor, a wafer was uniformly doped by arsenic implantation with an energy of 3 keV and a dose of 5 × 10^13^ cm^−2^, followed by rapid thermal annealing at 1000 °C for 10 sec. To uniformly spread dopants throughout the entire silicon on insulator, additional furnace annealing at 1000 °C for 10 hour was performed in a N_2_ ambient. A dumbbell-shape active region was defined by a standard lithography process. Device isolation was achieved by removing the silicon on insulator in the non-active region through a dry etching process. HfO_2_ was deposited on the silicon channel region for passivation. A Ni/Al stack was deposited to form a top gate electrode. The high work function of nickel helps to deplete the highly-doped silicon channel. The source-drain contact was made by aluminum deposition. Post-metallization annealing was carried out at 410 °C for 30 minutes in a 10% H_2_ ambient to lower the interface state density.

### Device characterization and measurement

The fabricated back-gated junctionless transistor was measured using a semiconductor parameter analyzer and a probe station. To characterize the mobility of the junctionless transistors, the field effect mobility was calculated from the conventional trans-conductance method, using the equation 
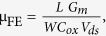
 where *L* and *W* are the channel length and width, respectively, and *G*_*m*_ is the trans-conductance, *C*_*ox*_ the gate capacitance, and *V*_*ds*_ the drain-to-source voltage. Atomic force microscopy, NX10 model manufactured by Park Systems, was used to characterize the surface roughness of the silicon with a new tip. The XY resolution is 0.05 nm and Z resolution is 0.015 nm. A scan area of 10 μm × 10 μm was analyzed using the XEI program to assess the RMS surface roughness, height histogram, 1D/2D power spectral density, and fractal dimensions. The fractal dimension was calculated by the triangulation method with a linear interpolation.

## Additional Information

**How to cite this article**: Kim, S.-Y. *et al*. Valley-engineered ultra-thin silicon for high-performance junctionless transistors. *Sci. Rep.* **6**, 29354; doi: 10.1038/srep29354 (2016).

## Supplementary Material

Supplementary Information
