# Supplementary material for: Valley-engineered ultra-thin silicon for high-performance junctionless transistors
Source: Sci Rep. 2016 Jul 8;6:29354. doi: 10.1038/srep29354 (PMC4937383; doi:10.1038/srep29354)
Supplement: Supplementary Information [file srep29354-s1.pdf]

Valley-engineered ultra-thin silicon for high-performance junctionless transistors

Seung-Yoon Kim[1], Sung-Yool Choi[1], Wan Sik Hwang[2], and Byung Jin Cho[1*]

[1]*School of Electrical Engineering, KAIST, Daejeon, 305-338 Korea,*

[2]*Department of Materials Engineering, Korea Aerospace University, Gyeonggi-do, 412-791, Korea,*

1. Reason for decision of minimum silicon thickness

The thinnest silicon thickness we fabricated was 2.5 nm. It was possible to thin down silicon more aggressively. However, if the thickness is below 2 nm, the edge effect which dopants or carriers hardly exist near edge becomes prominent.[S1] Then, the device would not be operated properly. Since the transport mechanism of junctionless transistors is based on carriers from doped atoms, this edge effect should be taken into account.

2. Calculating sub-band separation

Calculating sub-band separation is important to confirm valley engineering of silicon. If the sub-band separation is low, the quantum confinement effect would not be shown at room

temperature. Sub-band separation is a surface potential difference between two peaks when the derivative of conductance oscillation is zero. Since the surface potential could be affected by many device parameters such as gate capacitance, channel doping concentration, and gate material, the conversion equation from the gate voltage to the surface potential should incorporate all the values that could affect. Therefore, the following equation was used to calculate surface potential in conventional MOSFET with a polysilicon gate.[S2]

$$\varphi_s = \left(\sqrt{\frac{2qN_A\varepsilon_{Si}}{C_{ox}^2} + 4\left(1 + \frac{N_A}{N_{PG}}\right)(V_G - V_{FB})} - \frac{\sqrt{2qN_A\varepsilon_{Si}}}{C_{ox}} \right)^2 \cdot \frac{1}{4}\left(\frac{N_A}{N_{PG}}\right)^{-2}$$

The calculated values from this equation were presented in Figure 4d of the main text, where q: quantity of electric charge, N_A: channel doping concentration, ε_{si} : permittivity of silicon, C_{ox}: gate capacitance, and N_{PG}: polysilicon gate doping concentration.

In order to solve this equation for calculating the sub-band separation, the average inter-peak voltage was replaced by V_G-V_{FB}. The calculated sub-band separation, which indicates valley splitting, shows similar trend with conduction band variation in ultra-thin-body silicon.[S3] It is confirmed that the valleys split more, as the thickness of silicon nano-membrane decreases. The amount of valley splitting is 70.2 meV when there is 2.5 nm of silicon, whose energy is around 3 kT, where k: Boltzmann constant and T: absolute temperature. Other devices show ~ 2 kT of valley splitting which is large enough to reveal RTQCE.

3. TEM and strain analysis

Accurate measurement of the thickness of the silicon nano-membrane and strain analysis were performed by HR-TEM. The HR-TEM was a Titan double cross-sectional corrected TEM whose resolution was point resolution of 0.08 nm and both information limit and STEM resolution of 0.07 nm. We used accelerating voltage 300 kV. In order to analyze strain in the silicon nano-membrane by measuring atomic distance, a high-resolution TEM image is required. When performing simple Fast Fourier Transform (FFT) and masking, which were explained in Figures 2a to 2c in the main manuscript, it was impossible to obtain re-constructed an image with high resolution. Figure S1a shows a TEM image of a 2.5 nm thick device. As it can be seen, it is not only difficult to measure the atomic distance from the original TEM data, but also the measured data may not be reliable. Therefore, we adopted inverse FFT technique. Through the inverse FFT with applying a mask, atomic arrangement was clearly re-constructed as can be seen in Figure S1b.

4. AFM analysis - 2D power spectral density

The silicon surface after iterations of thinning process was analyzed, showing low RMS surface roughness and fractal dimension close to two. Figure S2a shows 3D tilted view of AFM image which was performed on a thinned top silicon surface of a SOI wafer over a 10 μm × 10 μm area. The silicon surface has extremely flat surface with several hundred picometer roughness at maximum.

2D power spectral density (PSD) analysis was performed to characterize the silicon surface. The RMS roughness and the fractal dimension decrease as thinning process is repeated. Such trend was confirmed in Figure S2b. In the plot of 2D PSD with respect to spatial frequency, the integration of the region below the data point line is proportional to the square of the RMS roughness. Two different polysilicon samples, deposited by low pressure chemical vapor

deposition, were prepared for comparison purpose. The 2D PSD of silicon was much smaller than that of polysilicon.

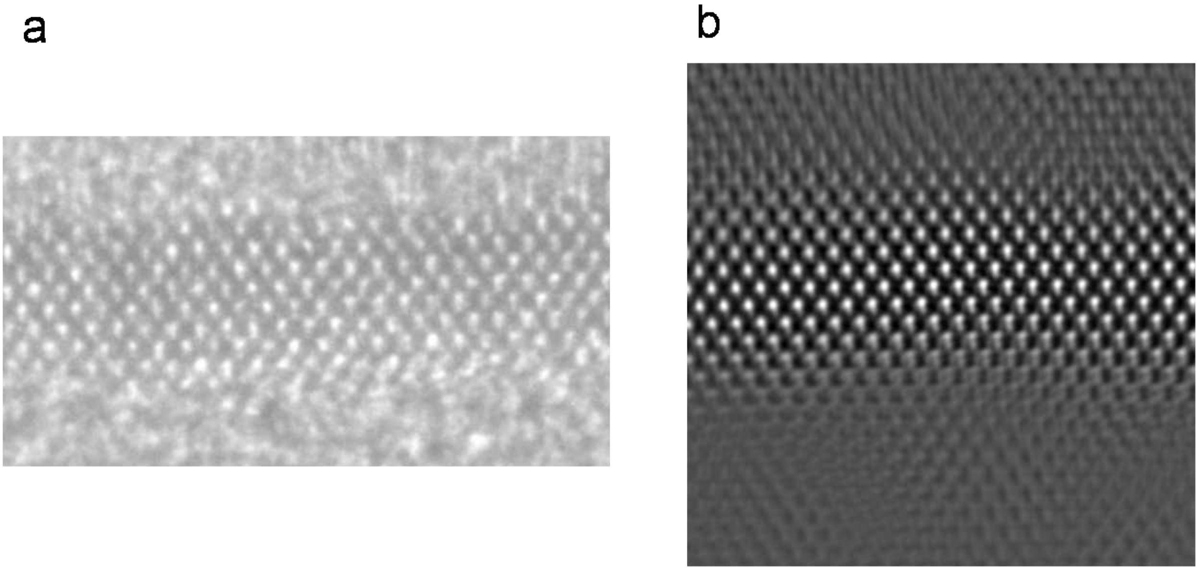

Figure S1. (a) Original TEM image of 2.5 nm thick silicon. (b) Reconstructed 2.5 nm thick silicon image through inverse FFT process.

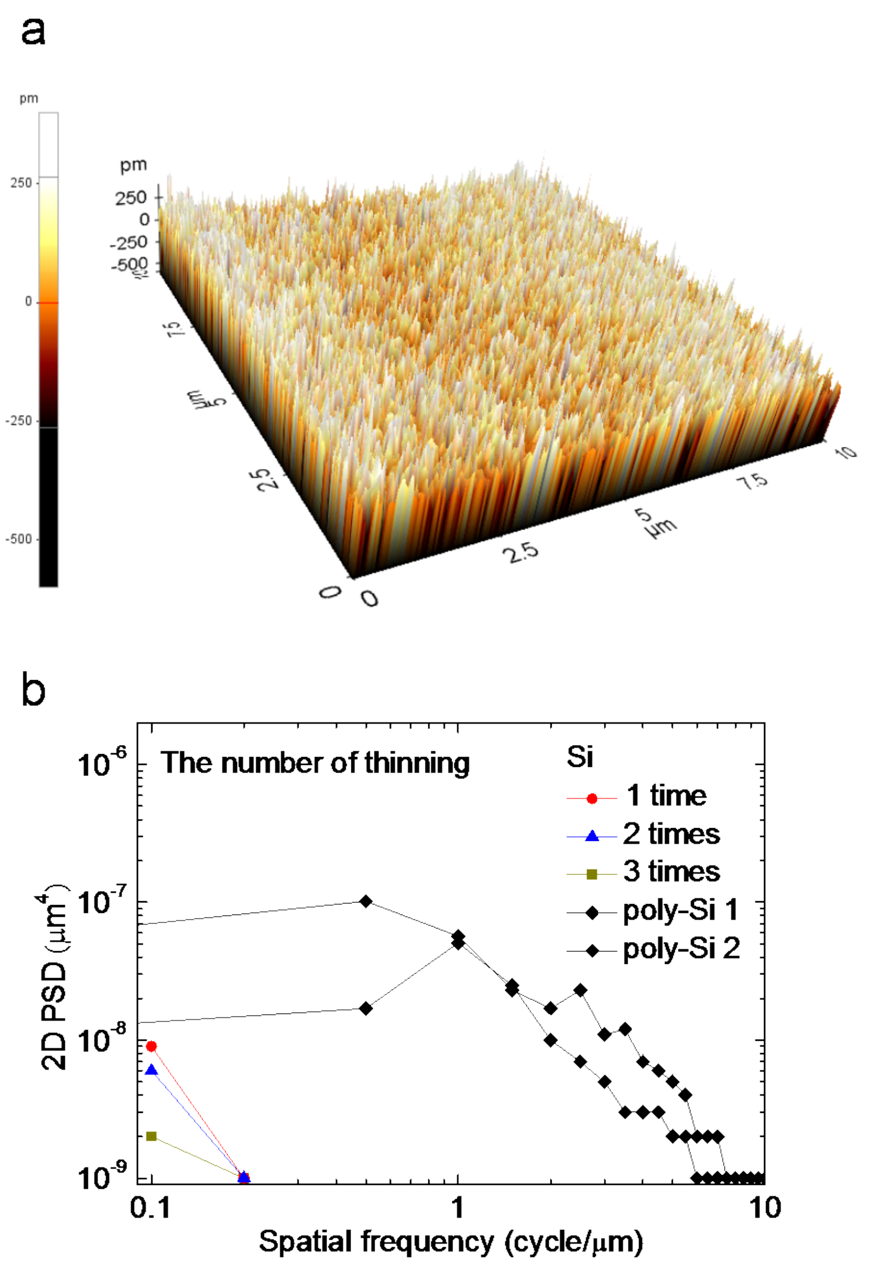

Figure S2. (a) Three-dimensional view of a three-times-thinned SOI sample that has 2.5 nm thick top silicon. Surface roughness characterization of ultra-thin silicon was conducted by non-contact AFM. (b) 2D power spectral density with respect to spatial frequency shows well-controlled atomic scale roughness and also the data is detected at low spatial frequency only. The integration of the region below the data point line is proportional to the square of the RMS roughness. The tendency of 2D PSD is also well matched to that of the height histogram. 2D PSD of two different polysilicon samples was also measured for comparison purpose.

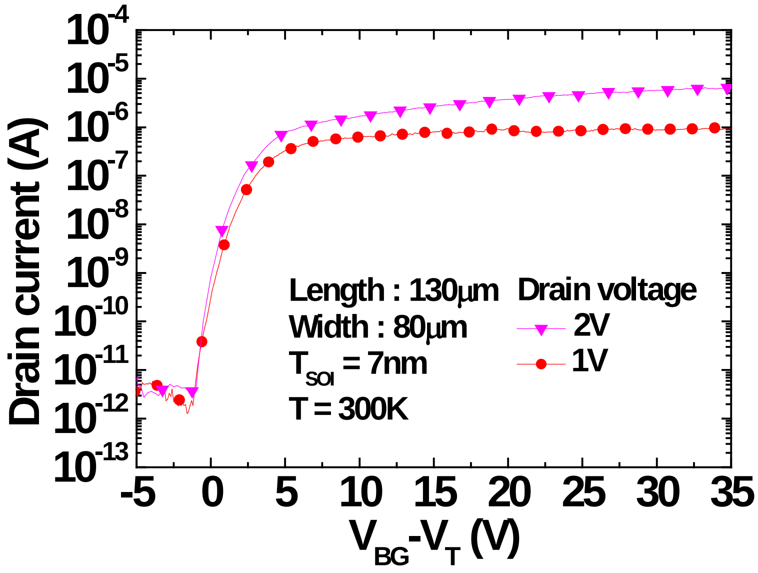

Figure S3. Transfer characteristic of a 7nm-thick silicon channel device in the log I_d vs V_g for the drain voltage of 1 V and 2 V. The device shows the on/off ratio of over 10^5 when the drain voltage = 1 V.

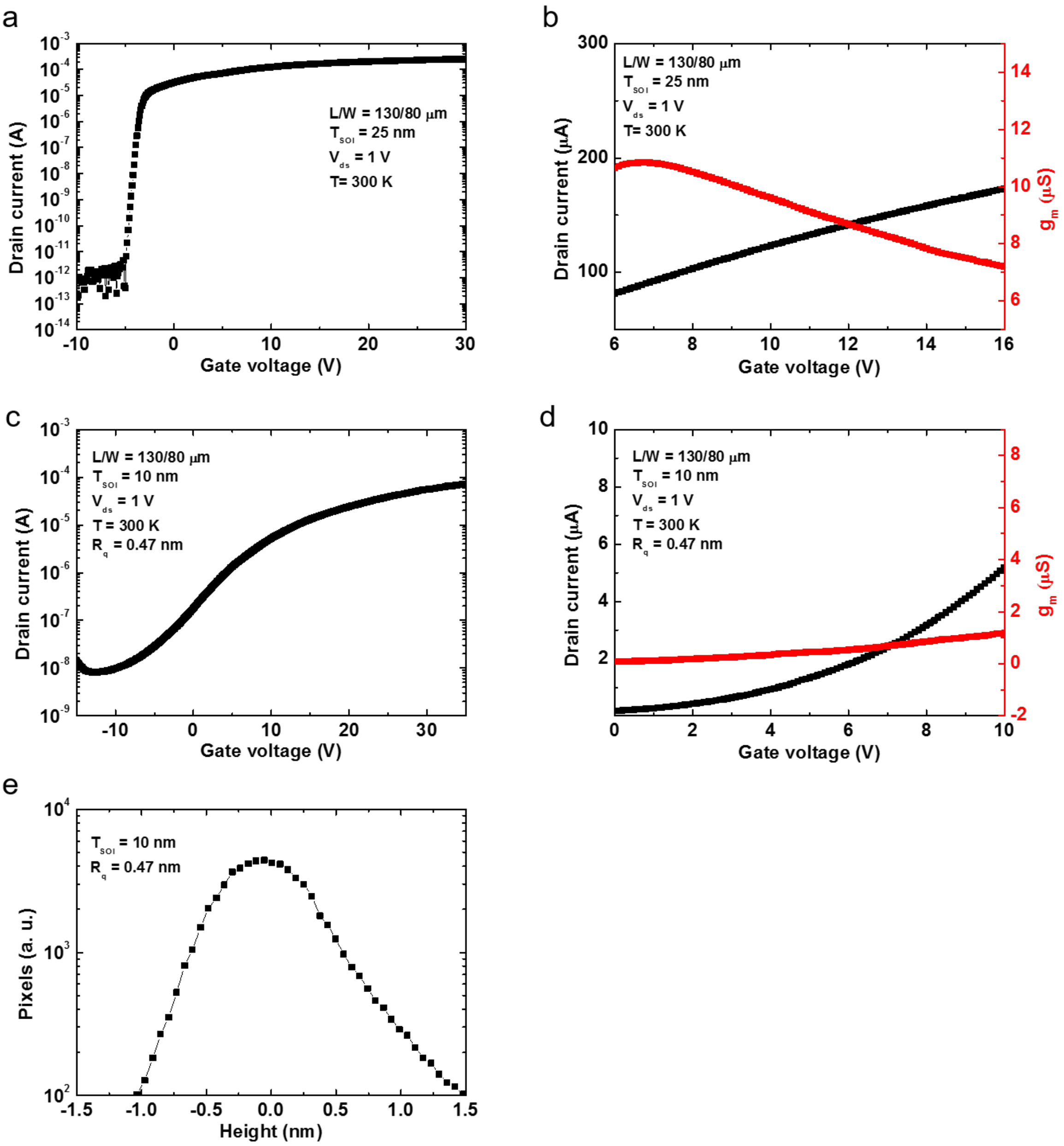

Figure S4. Silicon channel devices without RTQCE. (a) Transfer characteristic of 25 nm thick silicon channel device over a full sweep range of the gate voltage which does not shows RTQCE. (b) Enlarged scale of the transfer curve in (a). Transconductance of the device does not show any oscillation. (c) Transfer characteristic of 10 nm thick silicon channel device whose silicon RMS roughness of 0.47 nm does not show RTQCE over a full sweep range of the gate voltage. (d) Enlarged scale of the transfer curve in (c). Transconductance of the device does not show any oscillation. (e) Height histogram of 10 nm thick silicon film whose RMS roughness is 0.47 nm.

Supplementary references

S1. Rudenko, T. et al. Mobility enhancement effect in heavily doped junctionless nanowire silicon-on-insulator metal-oxide-semiconductor field-effect transistors. *Appl. Phys. Lett.* **101**, 213502 (2012).

S2. Kim, D. M. *Semiconductor engineering: Electrical characteristics of MOS structure* Ch. 11 (Kookmin univ., Hanbit media, 2011)

S3. Duarte, J. P., Kim, M-S., Choi, S-J. & Choi, Y-K. A compact model of quantum electron density at the subthreshold region for double-gate junctionless transistors. *IEEE Trans. Electron. Dev.* **59**, 1008–1012 (2012).

S4. Chanda, M., De, S. & Sarkar, C.K. Modeling of characteristic parameters for nano-scale junctionless double gate MOSFET considering quantum mechanical effect. *J. Comput Electron.* 1-8 (2014).
